# On Quackery

**Published:** 1862-07

**Authors:** W. H. Matchett

**Affiliations:** Ithaca. O.


On Quackery.—*By W. H. Matchett, M. D., Ithaca. O.*—In the early part of the year 1825 the light of the sun first beamed upon the head of our friend Quidnunc, the character we will introduce to you (as the novel writers say) as the hero of the following pages.

Poor Quid was an unfortunate child, for his earliest recollections were of misery and sufferings from all manner of infantile diseases, such as measles, mumps, whooping cough, etc., which followed each other in quick succession. Then the various prescriptions the poor boy was compelled to take for his many ailments caused him to wonder where all the knowledge came from to find out all about the virtue of the many things used by the old granny woman, who was his constant medical attendant.

Granny E. was a very useful personage in the neighborhood. She attended all the births, and eased the pains in all the deaths; neither event could be well attended to without her—at least so thought the citizens of the village. If any mishap occurred, the cry was, "Run for Granny E.;" and she was always on hand, bearing on her arm a large basket, in which she carried her many remedies. The village children were in constant fear of the old lady, and if they saw her coming, they scampered off and hid themselves. They all knew her as far as they could see. She rode a poor, old gray horse, bearing upon her arm the veritable medicine basket. She wore a blue calico dress, with a shirstring waist; a white handkerchief, that was crossed upon her breast, and pinned down to the waist on each side; a large leghorn hat, or bonnet, the crown about eight inches deep, and the front at least twelve inches high, at an angle of about forty-five degrees to the crown.

Quid's first attack was the measles. Granny E. was sent for. She dosed him with various teas to "fetch them out," but to no effect; after some hours' trial, she shook her head in a knowing way, and said, "I must try my never-failing tea." So she off to the sheep pasture, and procured what she, in her technicalities, styled "sheep nannie," of which she made a strong decoction, and forced it down the unwilling gullet of the patient, then covered him up close and warm, and hung quilts around the bed to keep off the cool air. After some twenty-four hours, she had a fine crop of measles on Quid's skin, which was alone attributed to the mysterious virtues of the "sheep nannie tea."

Shortly after, Quid took the mumps. Granny E. was again in requisition, whereupon she recommended and prepared a poultice, procured in the pig-sty (Stercus Porcinia), fried in lard, and applied it to the boy's throat; and to prevent the mumps from "going down," as she said, she applied a tight belt to the waist, made of flax straw, taken in the rough. This was the principal treatment, and upon it Quid did well; and recovered only to be attacked with the whooping-cough. Dr. Granny's great remedy in that was a teaspoonful three times daily of Urina Virgineus. Upon this treatment, after some weeks, poor Quid was restored to moderate health. He had all the while, for weeks after, a tightness of breath and a wheesing, that was called by his medical attendant phthisic (asthma.) For this, her potent remedy was peculiar. She took Quid from his bed before sunrise, on the first day of May, and proceeded to the forest, without speaking a word, and upon the *north* side of an oak tree, just as high as the boy's head, she bored a hole, and taking a lock of hair, as near the crown as may be, she pegged it fast in the hole with a cedar plug, leaving the patient tiptoeing it until sunrise, when she severed the hair, and left it sticking in the hole, with the promise to the boy that his malady would leave as soon as the hair rotted and the hole grew up.

Quid's distemper still increased, until he was nigh suffocating for the want of breath; when old Granny E. was again sent for, who, after a close examination, came to the sage conclusion that he was "*liver-grown*," and had the "*go-backs*."

First, she cured for the "*go-backs*," by measuring with a hemp string three times the length of the patient; then put said string under the hinge of the gate, on the *north* side of the house. The cure was to be completed as soon as the swinging of the gate wore out the string. And for the "*liver-grown*" Quid was stripped to the skin, greased all over with lard, laid upon the table and kneaded as a baker kneads his dough—frequently pressing her knuckles and finger ends under the ribs with such force as to make him squeal for dear life, and beg to be let alone to die some other way. But no let up; Granny knew what was best, and she must proceed until the operation was complete, which was as often as the patient was years old; then take him by the heels, and give him as many shakes, with the head downwards. This whole process was repeated seven successive days, because there are seven years of plenty and seven of famine; also, seven *sabbatical days* spoken of in the Bible.

In the year 1831, Quid having attained his sixth year, and passed through the application and administration of such a variety of "never-failing" cures, without losing his life, his parents removed from the little village of M—, and settled in a new district, or settlement, in the backwoods of Ohio. Here the population was much scattered; so much so, that a distance of five miles in circuit was taken in the same neighborhood, and every one in the neighborhood knew what was transacting in the settlement—also, what was the condition of the people. Quid soon became an object of interest and solicitation to all the knowing and marvelous ones, who recommended their various cures. All were duly applied, for the anxious and fond mother was very solicitous for her poor invalid boy.

In this *new* country the place of a physician was supplied (as in the village) by an old granny, until the joyful news spread through the settlement that one of the settlers had been off on a visit, and came across the celebrated Dr. Thomson, of whom he bought a *patent*, and had actually set up shop as a "*patent doctor*."

This was hailed as a God-send; and all eyes were turned upon Quid as the first patient of *patent* Dr. K. After strong solicitations by the neighbors, the mother at last consented, and the Doctor was sent for. He came, and began to prepare his "stuff and fixtures," when the mother suggested to the Doctor to examine the patient, and see what was the matter. Dr. K. answered: "Oh, no matter, madam; no matter what the disease is. We *patent* doctors do all things by a reg'lar rule; we put 'em through a course of medicines—that is, we pukes 'em, and purges 'em; then we steams 'em, and sweats 'em. This brings on the alarming symptoms; and, if they gets through that, the disease is broke, no matter what it is. Now, madam, get me three tin cups; put on a kettle of water; and we will put him through a course of medicine."

In one cup he made a decoction of lobelia; in another a solution of saleratus; in another a decoction of May apple (Podophyllum Peltatum.) About one-half pint of the lobelia was given poor Quid, followed by a like amount of the solution of saleratus; then, after taking a quantity of warm, salty water, the order of nature was reversed, and the contents of Quid's stomach was brought forth. After repeating this process to the satisfaction of the "*patent doctor*," who would exclaim at each emesis, "*what wicious stuff! what wicious stuff!*" he administered the May apple to turn it downwards—which had the desired effect in time to suit the Doctor's notion. He then prepared for the last part of the course, which was the steaming operation. This was accomplished by means of long tin tubes, extending from a vessel of boiling water in the fire place to the bed—the steam pouring under the bed-clothes, and coming so hot on the patient as to remove the skin with which it came in contact. It did not take long, after the previous *gentle* usage, to bring on the *alarm*; and so fearful was it, that all in the house, even the Doctor himself, thought the boy dying. The Doctor gathered up his traps, and had pressing business elsewhere. He rode fast and faster, no doubt feeling better as he placed distance between him and the victim of his patent treatment. Quid's mother, more firm than the rest, maintained her self-possession; and by her tender, motherly care—chafing the palms, and sponging the limbs, etc.—succeeded, at last, in resuscitating her child. She was a woman of some sense, and declared that "no more *up-start* doctors or conjuring old women should try any more of their *cures*, or *put 'em through* any more *courses* of their treatment."

After this, Quid was subjected to no more *patent doctoring*. But his malady was none the better; but by a careful regimen, the paroxysms diminished in frequency, until, at about the age of twelve, they ceased entirely.

Quid had an inquiring, quizzical turn of mind; and being diseased, was not able to do much fatiguing labor. He spent much time with books. The laws of nature, and the natural sciences as taught in the nursery books, and also in books for more mature years, as Comstock's Philosophy and Chemistry, Lincoln's Botany, Cutter's Anatomy and Physiology, etc., were his constant companions.

It was his mother's desire to educate him for the ministry; but the natural bent of the boy's mind caused him to choose —after arriving at a suitable age, and having passed through an academic course of study—that profession for life that would comport with his taste for the physical sciences. Hence, he chose medicine. Not medicine as taught and understood by the patent doctors; nor medicine fenced in by any one sectional idea; but that broad, comprehensive profession of medicine that includes, and even demands of its votaries, investigation in all departments of the natural sciences, from the chemical combinations and atomic unions of the *universe*—including the animal, mineral, and vegetable economy—even to the topographic and hydrographic knowledge of the earth.

For five years, under the direction of a preceptor who quizzed him closely, Quid pored over the large volumes on anatomy, physiology, chemistry, botany, therapeutics, surgery, obstetrics, etc., when he was considered by his preceptor far enough advanced to attend a course of lectures; which he did, in one of the best schools in the State. The next winter he attended another school, where he paid especial attention to dissections, making preparations for his cabinet. In the spring the Faculty graduated him an M. D. He returned to his preceptor, with whom he did business, under instruction, for one year, before he established himself, or thought he was qualified to assume the responsibilities of a practitioner of medicine.

Being located in practice, he was brought in contact with various men and minds in the profession. But what puzzled him most was to know how any one possessing *any degree* of morality could leave the plow in the field, or the bench in the shops, and start out as a medical man, without making any preparation as to study—simply buying a *patent*, or an old rejected book of receipts, or falling heir to some old medical library, etc. Yet such is the fact, and Quid had to contend with a nest of just such pups. For instance: on a certain day, a fellow who a short time before exchanged the shoe-bench for a pair of pill-bags, came after Quid to go with him to see one of his patients. Quid asked what the matter was. The would-be doctor said: "In the first place, it was a case of heavy constipation, with spasmodic fits;" for this he gave him medicine which "acted like a charm." Went to see him next day, and "found him entirely well all over, except the left ear was stiff." For this, he had made a "compound concoction of sixteen different articles in a gallon of whisky, and gave him that;" "this limbered his ear, but now the blood is pouring from his nose." "Epistaxis," suggested Quid. "I don't know whether it is *epicactus*, or what the d—l it is; but I do know he will die, and that shortly, if there is not something done more than I can do to stop the blood."

One night, about midnight, Quid was called to make great haste to a patient supposed to be dying. After he arrived, he ascertained that a fellow who a short time before was employed as hostler in a city livery stable, but who, becoming tired of his occupation, bought a *patent*, or what is the same, the tickets of a certain Eclectic medical college, and set up for " doctoring "—had attempted to vomit her for a cold; but failing to produce the emetic action, the lobelia had produced the narcotic effect, "*brought on the alarm*," as Thomson would say. The doctor was at work manfully, with a large eight-ounce syringe, throwing water into the bowels! He said he had given her sixteen injections, but he "could not make it stick long enough to work off the puke."

Quid was attending a patient who had the erysipelas of the face and throat. The case assuming a serious form, a celebrated doctor from a distance must be called in counsel. He came, and after examining the patient, came to the sage conclusion that "the man had sore throat, because the palate of his mouth and the almonds of the ear was down!" The counsel assumed all responsibility, and instituted treatment by tying up a lock of hair on the top, or crown, of the head, passed a stick between the knot and skin, and twisted the lock of hair tightly; and at every turn of the stick, would strike a smart blow on the head, in front of the twist, to jar the dislocated organs in place again! This failed to give relief; and on the next day he used the same means, assisted by a lever down the throat, to *pry* them back to place. This was more than the patient could stand, and the counsel was dismissed, and the patient left in Quid's care.

Again: an aged lady was taken very badly with what proved to be her last illness; must have the counsel of a very celebrated magnetic doctor, who lived a distance of some thirty miles. He came; found all manner of fault with Quid's treatment, and instituted a different. Assuming great and mysterious power, he assured the friends the patient would be well enough the next day; that all that ailed her, the magnetic equilibrium was disturbed by Quid's calomel. He "would fetch it all right."

Taking a horse-shoe magnet, he marched three times around the room, striking the walls with it at the corners; then stroking the magnet down from the chin to the toes, in contact with the patient's skin, repeated three times; then making some mysterious gyrations in the air over the patient, the cure was completed. After receiving his fee of twenty-five dollars, he departed, still firmly declaring, "the patient will be well by morning." Well, so she was, for she died that night.

A lady, *enciente*, near ninth month, had continence, or retention of urine; sent for a *patent doctor* (or, as he styles himself, an Eclectic—the same thing as a patent intensified) to relieve her. He attempted to pass the catheter by the sense of touch, but failed; he exposed his patient, and tried again by the aid of sight, but with no better success. He then administered an emetic of lobelia, and at every effort at vomiting, as might be expected, the urine was voided uncontrollably in the bed. The doctor boasted his success in the management of the case; but, unfortunately for him, she needed the aid of a physician again in a few days, and she sent for——some one else.

Quid's near neighbor, who was celebrated as a conjurer, or cancer-curer by words, frequently received letters from patients at a distance, and as he could not read them himself, would get Quid to read for him. By this, he learned the great method of cure was to learn the exact name and age of the patient; then take little sticks of some peculiar kind of green wood, in number corresponding to the number of letters and figures in the name and age; and upon a certain morning, before sunrise, to deposit said sticks in a spring of running water, the cure to be completed when the sticks returned to dust. It was astonishing to see what droves of people, and from what distance, would resort to this illiterate old person for cures. If you attempted to reason with them about it, they would refer to cases cured by this mysterious process. Of the same class are the hosts of blood-stoppers and fire-blowers. Relics yet of the age of witchcraft!

Not only the ignorant, who play upon the caprices and marvelousness of the world, but intelligence and refinement, not scrupulously honest, will do the same. For instance: a celebrated, intelligent man of Quid's acquaintance has made a fortune by staying in his office, keeping it well adorned with curiosities of art and nature, and entertaining his numerous visitors with music. He assumes an air of great mystery, and prescribes for all diseases, no matter what, drops No. 1 and drops No. 2, alternately, followed by pills of assafœtida. The prescription lasts about four weeks. Quid remonstrated with him about his course; his answer was: "The world will be humbugged, and I may as well do it as any other person."

It is this mysterious or marvelous condition of the public mind that gives support to that nonsensical idea of Homœopathy. No thinking, reasoning mind can see anything but nonsense in it. The same idea that old Granny E. advanced, in her stated number of shakes and greasings to cure *liver-grown.* In Homœopathy they have a stated number of shakes and triturations, to increase the power and add potency to remedies! Granny E.'s nonsensical idea intensified; a last struggling effort of fanatics and charlatans to put a scientific dress on some old mysterious ideas that were rife in the days of the Salem witchcraft! Add spiritual potency and power to drugs by shakes, triturations, and dilutions!! What folly; but not more so than that oft-repeated "*Similia similibus curantur.*" Stick your hand in the fire to cure a burn; or swallow lead to cure colica pictonum!

Quid was an incog. observer of a serio-comical occurrence that exposed the ignorance of one, and the keen retort of another, of these would-be wise doctors. A certain drunken, rowdy fellow got into a difficulty with his neighbor, who threw a sharp stone and hit him on the temple, severing a small artery. A *patent* doctor was called to dress the wound. He piled on wet cloths to the thickness of two inches, to arrest the bleeding, but to no effect. The friends became alarmed; went for another *doctor*, who, seeing the nature of the cut, made pressure on the proximal extremity of the bleeding vessel, and, of course, stopped the hemorrhage immediately. The first doctor, astonished at the quick work, asked, "Did you say some words, Doctor?" "Yes," was the response. "Will you tell me what they are?" "Presently, I will." They dressed the wound and prepared to leave, when doctor No. 1 asked again what those words were. "I said, if you were not such a fool, you could stop blood as well as any one," replied doctor No. 2. Quid thought it, under the circumstances, a cutting but well-timed rebuke.

In contemplating the standing and qualifications of the *country* practitioners, Quid came to the conclusion that at least one-fourth of them began practice, even among those who claim to be Allopathic, without making suitable preparation as to study, and after entering the field of practice, they quit study altogether. Some, he found, after investigating their precedents, had failed in business of another character; got a few medical books; read from four to six months; perhaps spent a winter at some second-rate school, and established themselves as finished doctors; and to make the public think they know something, use the pronoun I with great emphasis in speaking of operations they *saw* performed in the clinics, as though they took part in them. Some even go so far as to claim to have been *hospital* physicians, when indeed and in truth they were only permitted, by virtue of a ticket, to spend two hours a week, as a spectator, in the lecture room of said institution. Such fellows assume to know much ; make use of a great many technicalities in describing anything, solely for the purpose of bamboozling the public in the support of their arrant humbuggery.

The mind that has spent but a *few* months with the text-books of medicine can not appreciate or even comprehend one-half of what is said in a course of lectures, for it is not sufficiently drilled to understand the technicalities made use of. Consequently, a course of lectures to a mind unprepared by previous study is thrown away; and to give such a one credit for a good knowledge of his profession is the same as giving the school-boy credit for a perfect knowledge of the alphabet, when he has only learned the *alpha* and *omega* of the same; or for a complete knowledge of all the lessons in his school-reader, when he has read only the first and last.

It is a rare thing for a man not drilled to study during a lengthened pupilage to study much after locating for practice. They spend most of their time on the corners, or in shops and public places, at their usual business of "gazing" about the great deeds done and great cures performed. They are much like Longfellow's "Iago," in the Song of Hiawatha :

> "Never heard he an adventure,
> But himself had met a greater;
> Never any deed of daring,
> But himself had done a bolder;
> Never any marvelous story,
> But himself could tell a stronger."

Now, gentlemen, let us dismiss our friend Quid—we know he but tells the experience of us all,—and contemplate for a time what we conceive to be the best method to get rid of this growing evil. I say *growing*, for assuredly it is not diminishing. What was a short lifetime ago confined to a few old ignorant women is now seized upon by men who claim respectability and learning, and have clothed the humbug in a glowing dress of high-sounding names, as Homœopathy, Hydropathy, Uriscopy, Eclecticism, etc.

I, as an individual, claim, and charge it upon the profession generally, that the success of these evils is chargeable to ourselves. We are not bold enough in denouncing impostors, for fear the public will charge us with jealousy—fearful of injuring our popularity. Many of us will even counsel with them; and as long as we do so, we are recogrizing them as equals—not raising them to a standard of respectability by any means, but lowering our character and the dignity of the profession to an equality with arrant humbuggery.

The statistics of our country show the longevity of this nation is diminishing, while in other countries, where sanitary regulations and medical practice are governed by law, it is increasing—the average duration of life is longer.

This certainly is not attributable to our climate, for it is conceded by philosophers of all countries to be mild and healthy. It can not be attributed to the water or food, for indeed we have both in abundance, and for purity the world knows not better. Then it must be chargeable alone to our loose sanitary regulations, or rather to the want of any regulations at all.

We, as a nation, are very particular indeed as to the mental culture of our children. In every county town there is a board of examiners who must first give a certificate of *qualification* before any one is permitted to teach in our public schools—administer to the mental training of the child. *This is the law.* and it is right. Yet any charlatan or mountebank is permitted to administer to the physical wants, upon which the mental power depends, and the law does not know anything about *his* qualification to do so. This is not as it should be; the public demands as much, yea, more protection in its physical than in its mental training, and we, as the guardians of the public health, should "cry aloud and cease not," until legislation make the necessary arrangements to accomplish the object.

The first step in this direction would be to have an institution endowed by the State for the support of the professors, as in the University of Michigan, so that the faculty will not have to depend upon the number of students and sale of tickets for a support. Then let *merit* or *qualification* alone be the price of matriculation, and let all candidates for graduation pass examination by a board of censors, also appointed by the State. A certificate of qualification should be obtained from this board by *all* who locate to practice medicine, irrespective of school, class, sect, or kind, or suffer a penalty.

Secondly. A physician should not assume the position of preceptor until he is satisfied, from personal examination or otherwise, that the applicant has at least a good English education. I will not contend that a student *must* be a linguist, yet it would be greatly to his advantage, and facilitate his study of medicine, to be able to read Latin, at least. The preceptor should require a written obligation from the student that he would not attempt to practice until he has spent at least *three* years in the office, or under instruction, and attended one full course of lectures in a respectable school of medicine ; and if it were extended to graduation, it would be still better.

Thirdly. Let all physicians of a country or district unite in a medical society, and take cognizance of the actions of the members ; and frown upon, or punish by censure or penalty, all departure from the rules thereof, or of the American Medical Association ; and it will be but a few years until such a thing as a six-month doctor or impostor—among regular physicians, at least—will be unknown.—*Cincinnati Lancet and Observer.*

